# Unpacking the psychiatric advance directive in low-resource settings: an exploratory qualitative study in Tamil Nadu, India

**DOI:** 10.1186/1752-4458-7-29

**Published:** 2013-12-26

**Authors:** Laura S Shields, Soumitra Pathare, Selina DM van Zelst, Sophie Dijkkamp, Lakshmi Narasimhan, Joske GF Bunders

**Affiliations:** 1 Athena Institute, VU University Amsterdam, Amsterdam, The Netherlands; 2 Law and Policy Action Lab, Centre for Mental Health Law and Policy, Indian Law Society, Pune, India; 3 The Banyan Academy for Leadership in Mental Health, Chennai, India

**Keywords:** Psychiatric advance directive, Decision-making, India, Community mental health

## Abstract

**Background:**

Psychiatric advance directives, a tool to document preferences for care in advance of decisional incapacity, have been shown to benefit persons with mental illness in a number of countries through improving medication adherence, reducing symptoms from escalating in a crisis, accelerating recovery, and enhancing service user autonomy. While concepts such as autonomy are important in a number of high-income country settings, it remains unclear whether tools like psychiatric advance directives are suitable in a different context. The recent introduction of the psychiatric advance directive into draft legislation in India prompts the question as to how feasible psychiatric advance directives are in the Indian context. The aim of this study is to explore the feasibility and utility of PADs in India, with a focus on the need for individual control over decision making and barriers to implementation, by exploring views of its central stakeholders, service users and carers.

**Methods:**

Qualitative semi-structured interviews (n = 51) with clients (n = 39) and carers (n = 12) seeking mental health treatment at outpatient clinics in urban and rural settings provided by a non-profit organisation in Tamil Nadu, India.

**Results:**

Clients engaged in a number of forms of decision-making (passive, active, and collaborative) depending on the situation and decision at hand, and had high levels of self-efficacy. Most clients and carers were unfamiliar with PADs, and while some clients felt it is important to have a say in treatment wishes, carers expressed concerns about service user capacity to make decisions. After completing PADs, clients reported an increase in self-efficacy and an increased desire to make decisions.

**Conclusions:**

The introduction of psychiatric advance directives in India appears to be associated with positive outcomes for some service users, however, there is a need to better understand how this tool can be adapted to better suit the care context in India and hold meaning and value for service users to complete.

## Background

Globally, the way we perceive disability is changing, from seeing persons with disabilities as objects of charity to subjects with rights, capable of claiming these rights and participating actively in society [1]. Article 12 of The Convention on the Rights of Persons with Disabilities (CRPD) (Equal Recognition before the Law) is representative of this paradigm shift, stressing that systems should shift from models of plenary guardianship to models of supported decision-making to enable persons with disabilities to exercise their legal capacity. Legal capacity includes access to the civil, political and juridical system, legal independence to speak on one’s behalf, autonomy to make choices, and support to make these decisions where necessary [1]. However, persons with disabilities are not always able to exercise their legal capacity, thus there is a need for tools to enable supported decision-making. Legal incapacity might arise because of various factors. Legal systems may create legal incapacity due to guardianship provisions for persons with mental illness, or legal incapacity might arise because of decisional incapacity and mental incapacity in the midst of an active phase of illness. Legal capacity should not be confused with mental capacity, which is the ability to understand incoming information, considering harms and benefits of making or abstaining from a decision, and the ability to communicate the decision to others [2,3]. The relationship between the two concepts is that the cognitive requirements for mental capacity are also needed to exercise legal capacity; however, someone with mental capacity might not be afforded the opportunity to exercise legal capacity.

Psychiatric Advance Directives (PADs) are a legal tool for recording and implementing preferences in advance of periods of decisional incapacity [4]. PAD’s can both provide instructions for future care and outline personal choices (e.g. finances and housing); preferences for care, and/or specify the appointment of a proxy decision maker (nominated representative). These preferences can be expressed independently or with support from facilitators (the latter case is referred to as a facilitated-PAD) including health professionals or peer support workers (e.g., see Swanson et al., 2006) [5]. Several advance planning tools exist for persons with mental illness (e.g. joint crisis plans, crisis cards, advance agreements) requiring various degrees of collaboration with a health professional [6]. Some of these tools are legally binding (similar to PADs); however, PADs are unique in that they do not necessarily require collaboration or agreement from professionals, thus seen as enabling choice in decision-making. In addition, PADs could potentially mitigate the risks of coercive treatments to persons with severe mental illness during vulnerable periods when their autonomy is most compromised.

PADs in high-income countries such as the US and UK are associated with restoring service user autonomy, as well as improved medication adherence, reducing escalation of symptoms in a crisis, increase satisfaction with treatment, accelerating recovery, and enhancing dialogue with health care professionals [4,7-9]. However, autonomy is not necessarily the most dominant principle in many low and middle-income countries (LMICs), where cultural emphasis centres more on reciprocity, family, community, and joint decision-making [10]. In many LMICs, for example, the family often assumes the role of decision-maker, instead of the individual, responsible for ongoing mental health care [11].

This is also the case in India, where mental health care often aims to do what is best for the family [11,12]. This is also embedded in India’s *Mental Health Act* of 1987, which recognises the importance of family as the primary decision-makers; however this system has been criticised as it leaves the potential for family members to abuse these decision-making powers [11]. Currently, a new Mental Health Care Bill (2013) has been cleared for parliamentary review. The revised Bill adopts a more rights-based approach to care and has made an explicit provision for PADs as a way to promote supported decision-making. The draft legislation provides for a PAD that can a) specify the type of treatment a service user may want b) the type of treatment a service user may not want and c) the person the service user wants to make decisions as a nominated representative (proxy decision maker). While the draft law in India could empower persons with mental illness to have more autonomy over decision-making, there are substantial barriers to overcome, most notably sparse finances and human resources as well as a lack of awareness among health care workers, families and service users about ethical frameworks, weakening the value of available tools for protection of human rights of persons with mental illness [11].

In this article, we describe initial experiences with a trial of PADs in India; a tool that previously has not been used in cultures that espouse family rights over individual decision- making rights. It can be anticipated that many barriers occur while implementing PADs, thus it is crucial to explore the perspectives of stakeholders to understand the feasibility and utility of PADs [13]. Therefore we specifically aim to explore the feasibility and utility of PADs in India, with a focus on the need for individual control over decision making and barriers to implementation, by analysing views of the central stakeholders, service users and carers. This aim raises several questions relevant to the Indian context. First, what underlying beliefs concerning decision-making do service users hold? Second, what do service users and carers think about the concept of PADs? Third, can a tool originally developed as a way to exercise autonomy be valued and used in the Indian context? Fourth, does completing a PAD have any impact for service users?

## Methods

### Design

This qualitative study was part of a larger study assessing the feasibility and utility of PADs India, including the process and content of PADs. This study presents the results from interviews conducted before and after completing a PAD with clients and carers.

### Sample and setting

#### Study location

This study took place in Tamil Nadu, a state in Southern India. There are a number of public government funded mental health services at the tertiary level, and the District Mental Health Programme (DMHP) is implemented in several districts throughout the state, providing mental health care in the community. A number of private providers (including non-governmental organisations) provide care across the state. Clients in this study were existing outpatients registered with a non-profit mental health services organization (The Banyan) that operates in Chennai and surrounding regions. The Banyan provides a full range of services such as prevention, community-based care, tertiary care, rehabilitation and re-integration, community awareness, and policy advocacy. The Banyan has two flagship programmes: an Urban Mental Health Programme (UMHP) with several sites in Chennai, and a Rural Mental Health Programme (RMHP) based in Kovalam, a fishing village approximately 40 kilometres from Chennai.

#### Recruitment and sample

Clients are current outpatients (receiving combinations of pharmacotherapy, psychotherapy, and social care) from three sites within the UMHP and RMHP (two urban clinic sites, one rural clinic site). Clients were consecutively screened during outpatient clinic times for selection to participate in the study, and asked by a health worker if they would like to participate in an interview, and complete a psychiatric advance directive after being briefed by the health worker.

The sample was stratified by gender and location (i.e. equal numbers of clients living in urban and rural settings). This was done to ensure that clients living in rural areas and women (who are often underrepresented) were included in the study. Clients attending outpatient clinics during a 3-month period were screened and clients were chosen using a table with sex (male and female) and living situation (urban and rural) to balance participant selection. Clients were included in the study if they were diagnosed with a severe mental illness by the practicing psychiatrists at The Banyan (with or without active phase symptoms) and the client was able to understand, speak, and or/write in Tamil or English. Participants were excluded if they had a diagnosis of mental retardation, organic psychosis, or comorbid alcohol and/or substance abuse, as well as any person acutely ill to the extent that it would be unethical to have them participate in the study. Exclusion criteria were applied by consulting with the practicing psychiatrists at The Banyan and client records. In the results, we use the term clients to reflect the study sample accessing services at The Banyan, whereas the term service user is used throughout the remainder of the text.

#### Training and procedure

A training programme was conducted prior to this study to train PAD facilitators working at the implementing service organisation. The participants of the training programme were all health workers at The Banyan (case managers, social workers, psychologists, nurses, psychiatrists). These facilitators assisted in PAD completion, if requested by the client. The focus of the training/education programme was on i) providing the basics of a PAD and its relevance for clients and staff ii) providing guidance on strategies to facilitate PAD’s iii) to use examples and vignettes to depict possible ways of facilitating PADs, what completed PADs look like, and how they are used. The training also had components focusing on using PADs in a crisis situation and the use of PADs in future services provided by The Banyan. This training was expected to equip the facilitator (health worker) to introduce the PAD to the client, communicate its potential value to the client, and guide the client through the process of writing a PAD during the outpatient consultation or at an agreed upon time. The guide for facilitating a PAD can be found in Additional file 1.

### Data collection

A total of 51 semi-structured interviews took place between March and June 2013, carried out by 2 masters-level trained researchers. Both researchers received training on PADs by a psychiatrist (SP), and additional, context specific, qualitative research training. Interview guides and concepts to be used in the interviews were translated from English to Tamil and back translated to English to ensure concepts retained the same meaning during translation. A translator carried out interviews in Tamil, which were audio recorded, and translated into English. Interviews were carried out in three phases with different samples.

Prior to introducing PADs, 26 interviews were conducted with clients to explore notions of decision-making (Part 1 of this study). Then, clients and carers (n = 25) were interviewed on their knowledge and attitudes towards PADs (Part 2). Third, after completing a PAD, 18 clients (of 26 interviewed in Part 1) were interviewed on the perceived impact of PADs. As the other clients were not available for a follow-up interview (n = 8) post-PAD completion, it was only possible to interview 18 clients. Interviews post-PAD completion was carried between 1–8 weeks after PAD completion to allow time for reflection (depending on the client’s next scheduled appointment at the outpatient clinic). Interviews prior to PAD-completion explored whether clients: felt able to make decisions for themselves (and what form these decisions took), had goals, felt in control over situations occurring in their lives and their perceived ability to discuss treatment preferences with others (e.g. family members). In addition, clients and carers were asked about their attitudes to PADs through the following topics: whether one should have a say in their care; whether the client had ever been asked about their care preferences; awareness of the existence of PADs, the potential desire to use a PAD and perceived value of a PAD, and perceived adherence to PADs from care organisations. Post-PAD completion interviews with clients focused on reflections of completing a PAD, whether the PAD elicited feelings of greater decisional control, ability to express preferences, and finally, whether they would recommend PADs to others to complete.

### Ethical considerations

Local ethical approval was obtained for this study from the external research review committee for the Banyan. Consent to participate in the interviews and to complete a PAD was obtained and recorded from every participant either through signature on informed consent forms, or by thumbprint (See Additional files 2 and 3). To ensure confidentiality during the data analysis, names were replaced with unique ID codes. Furthermore, identifying factors associated with the interviewees were omitted from the data analysis and from this paper. Patient records and completed PADs were only accessible for staff working at the the Banyan and the core research team, and were locked and stored in a secure storage unit at the tertiary care centre run by The Banyan.

### Analysis

For clients, basic demographic and clinical characteristics were collected and tabulated (age, sex, rural/urban living situation, marital status). Basic demographic characteristics (age, sex, relation to relative with a mental illness, urban/rural location) were collected for carers. These demographics were available through client records maintained by the organisation.

For the qualitative data we used a thematic analysis approach [14]. The coding process consisted of several stages. Stage one involved two researchers familiarising themselves with the interview data. Stage two entailed two researchers independently coding the data inductively, creating a list of emergent codes. Codes were generated, collated, and refined into themes relevant to the research questions. A third researcher independently coded the interviews as an additional quality check. Disagreements were resolved through discussion or involvement of a fourth researcher on the project team. All interviews were analysed in MaxQDA, version 11 [15].

## Results

The majority of clients were between 18–49 years of age, while the majority of the carers were over the age of 60). Carers were either a parent (n = 7) spouse (n = 3) or son/daughter of a parent with mental illness (n = 2). Males and females, and urban and rural living contexts were equally represented across clients and carers (see Table 1). In total, there were 8 dropouts (n = 5 dropouts between the pre-PAD interview and post-PAD interview, who did not write a PAD), and n = 3 who completed the PAD but did not complete the post-PAD interview).

**Table 1:** Characteristics of clients (n = 39) and carers (n = 12) participating in interviews

**Characteristic**	**Clients (N = 39)**	**Carers (N = 12)**
Sex (male)		
Male	18	6
Female	21	6
Age group		
18–29	8	1
30–39	9	0
40–49	11	4
50–60	5	1
60+	1	6
Marital Status*		
Unmarried	10	
Married	13	
Educational status**		
Low	15	
High	3	
Living environment		
Rural	19	6
Urban	20	6

*Note*: *missing values *n =* 3; **missing values *n =* 20; values for marital status and educational status for carers not available.

### Notions of decision-making

PADs aim to enhance control over decisions and choices related to daily life and health care. Prior to the introduction of PADs, clients reflected on their ability to make decisions in their lives. From the interviews, a number of themes emerged: self-efficacy, control over circumstances, and types of decisions made.

Clients conceptualised self-efficacy as setting goals and attaining them. All clients have had periods in their lives where they experience high levels of self-efficacy regarding their personal lives and medical needs i.e. feeling committed to achieving a particular goal, capable of making decisions, and influencing their circumstances. In addition, all clients, irrespective of gender, felt motivated about attaining a state of good health, “getting well”, and, recovery. Clients who felt highly self-efficacious were also more likely to feel that they possessed the ability to make decisions independently and achieve goals.

*“I think I can accomplish any goals I have in life, I have confidence in myself that I can achieve them”* [ID 19, Female, Client].

Yet, a number of clients (n = 13) women (n = 8) and those living in rural areas in particular, felt they had low levels of self-efficacy, especially regarding setting individual goals. Women often set goals driven by family matters, such as ensuring family cohesion, well-being, and taking care of educational and marriage arrangements for their children. Seven clients, mostly women, indicated that they had never thought of setting individual goals. Nine clients also talked about “leaving behind” goals or decisions they wanted to pursue, largely due to pressure from family, or symptoms from their mental illness. In practice, this was described as having to leave a job due to recurrent symptoms or relapse, not having funds to send their child to school, or wanting to pursue a career but having a career choice decided for them by family members. It was articulated that goals only pertained to “larger” goals, smaller, more mundane attainable goals were not considered.

*“I have no goals in my life. When I was young I had a lot of goals..I wanted to get my own house and I wanted to be very happy. But now I do not have any goals of that sort, the only goal I have is to get my son married. There are no other goals, I just left all that I had before”* [ID 12, Female, Client].

The degree to which clients made decisions independently was again context-specific, where, across the interviews, the type of decision appeared to determine the level of required support or consultation with others. Clients described engaging in three forms of decision making: active (operationalised as making decisions independently), passive (choosing to hand over decision-making powers to someone else for a particular decision) and collaborative forms of decision-making (joint decision-making).

*“I make decisions for myself, and do not depend on anybody else. Although I discuss decisions with my sons, I make decisions for myself. I got my sons and daughter educated, got them a job, and got them married. This is a very big achievement and very important decisions I’ve made in my life”* [ID 11, Female, Client].

*“I usually make decisions for myself, but in the middle of my life when I was in a psychological depression, I approached another person for decisions and suggestions. Usually, I make my own decisions and it has always been like this for me. When I make decisions, and I am concerned about whether my choices are right or wrong, then I ask for suggestions from my friends and family”* [ID 8, Male, Client].

*“ I do not take any kind of decisions individually, I consult with my husband and we make decisions together”* [ID 14, Female, Client].

Five clients (all women) felt unable to make decisions independently or jointly, often due to their guardians (families) making decisions on their behalf without consulting them. Barriers to decision-making were attributed to dominant family members, or distress that impeded the ability to make decisions clearly. All clients described that there were few perceived barriers to sharing their preferences for care with family and/or health care professionals they frequently consulted with at the Banyan. Similarly, all clients described moments when they negotiated aspects of their care or daily living activities with others. This involved consulting with health care professionals, family or friends about their personal problems they were experiencing, particularly in relation to their mental illness (e.g. side effects from medication, tensions with the health care worker, working conditions).

*“I take the courage to talk with others and tell the doctor what I am feeling, even when I feel like I can’t. I take courage to do this because I think that I need treatment to be fine, so I discuss everything”* [ID 14, Female, Client].

Fourteen clients (of 26) currently felt in control over circumstances (described as being in control over thoughts, emotions, and reactions), especially over their health and treatment adherence, both at present and in the future. Despite accounts of low self-esteem and goal-setting behaviour, women described feeling in control over circumstances. Lack of control was attributed to the internal factors arising from their mental illness, either related to symptoms, or lack of confidence at the time to control emotions. External factors were also discussed as leading to loss of control, such as low-income or unanticipated life events (losing property or money). Poverty led many (n = 17) to feel not in control of resources (food, transportation, resources required to get a job, study, marry), contributed to low self-esteem, and made it difficult to envision goals. This led clients to feel sad and tense. A minority of clients (n = 2) felt no necessity to control situations, believing that it is not possible to control their fate:

*“I wanted to get my own house for myself, but then situations were such that things did not happen the way I wanted. Situations were very bad, now I have lost a lot of money because of that, and the situation was out of my control. I still had the courage to make decisions, but I still did not feel that the situation was in my control”* [ID 12, Female, Client].

Clients described feeling more stable and in control when receiving care (e.g. medication or counselling), as it equipped them with a better understanding of the situations occurring in their lives, and stabilised their emotions:

*“Now, since I am receiving treatment, I feel better and able to control situations. I used to feel very angry, upset and very depressed… Had I taken treatment at the time [of an incident from his past] I could have controlled the situation and handled it better”* [ID 23, Male, Client].

### What are the perceptions of PADs among clients and carers (families)?

#### Awareness of PADs

Clients were either unaware of PADs or its use, or were unable to describe and interpret the purpose of the PAD. Yet, all clients described situations in which they had been asked about their treatment wishes (for example, if they would like to see a particular doctor, or if they liked their medication).

*“I’ve been asked about what medicine I prefer to take. I’ve been asked this every week, whenever I come here [to the outpatient clinic]”* [ID 27, Female, Client].

Similarly, 11 (of 12) carers were largely unaware of the existence of PADs*,* with only one carer saying she had heard about PADs during a monthly meeting for clients and carers at the Banyan.

#### Attitudes towards PADs

Clients were asked about whether they felt they should have a say in their treatment and whether they would hypothetically like to have a PAD. Ten clients were ambiguous in their responses, with only one client explicitly liked the idea of having a PAD:

*“It would be better if the doctor asked me, and I have a say. I think that since I have an education, I can give projections on how I feel and how and what kind of treatment I prefer to have, but I have not been asked on that. The doctor doesn’t ask me, but I think the doctor should ask”* [ID 32, Female, Client].

Two clients stated PADs would be helpful both for treatment plans and as a way to encourage empowerment. However, they indicated that it is ultimately the doctors’ decision to determine treatment, and did not feel that one “should” have a say in their own treatment:

*“How can I decide on the treatment? The doctor has to decide that. The doctor knows whatever he is doing and I do not have a problem with it and I’m fine with it. I think it’s fine even if I am not asked about my opinion about my treatment wishes”* [ID 36, Female, Client].

### Carer views on service user capacity

Only one carer felt her mother should make decisions about her own treatment. This carer attributed her mother’s capacity to make independent decisions to her recovery. In the past, her mother was unable to make independent decisions due to the severity of her mental illness; however, since recovery, her decision-making abilities evolved:

*“I want my mother to fill in the PAD document, because if she can express her wishes better then it’s good for her. It can help her in recovery. Because my mother has already worked as a nurse, she knows better about injections and treatment and all that. She knows better about taking treatment, so it is better if she is the one who fills the PAD document”* [ID 5, Female, Carer].

Six carers were uncomfortable with the idea of their family member having a PAD and having a say in their treatment. Two primary reasons for this emerged from the interviews: Carers felt PADs were not useful and would not bring any additional sense of control to clients, or carers felt their family member had limited or no decision-making capacity. Other carers articulated that clients could make smaller decisions independently but were dependent on others for larger decisions:

*“I don’t think she [client] should be given a say, because she will not know what will happen in the future and she will not be able to decide on what kind of treatment would be good for her in the future”* [ID 3, Male, Carer].

*“Even the small decisions, he [client] consults with me [mother], and he depends on me whether he wants to go out or not, so he is not able to take decisions individually. He is always dependent on me”* [ID 2, Female, Carer].

*“She’s not capable at all [of writing a PAD]. She was once [capable], but now it’s just not as good as it was some five or six years back. I don’t think she would be able to express her wishes, or that she’s capable… Her ability is not to that extent.”* [ID 1, Male, Carer].

Interestingly, while clients themselves felt able to make decisions, especially about future care and treatment, it appears that carers did not always share this view. However, seven carers felt capable to assist their relative/friend in writing a PAD. Nonetheless, carers indicated they require support from health care staff as they have limited knowledge on the illness and treatment options.

### Perceptions about health care workers adhering to PADs

Most of the clients (9 of 13) and carers (9 of 12) receiving care at The Banyan expected health workers to follow wishes and preferences, if documented. However, when asked to envision whether health care workers in other services (outside The Banyan) would adhere, the reaction was less positive, as there was a general sense of mistrust (based on previous negative experiences) in other care providers adhering to treatment wishes.

### Client perceptions after PAD completion

Of the 18 clients interviewed after completing a PAD, 13 were able to articulate their treatment decisions independently without support from carers. Sixteen (of 18 clients interviewed post-PAD completion) felt that the PAD helped improve their abilities to make decisions about future treatment and feel more self-efficacious, and felt that the process of developing a PAD was easy. Sixteen clients articulated that they were able to openly express their preferences to the facilitator, and knew what kind of care they wanted to receive:

*“I was able to express my preferences freely, I expressed everything, whatever medicines or tablets or counselling I am taking, or would like to take, freely to the counsellor. Before filling the PAD I did not feel this way, but during filling out the PAD I felt it was important because I felt that others have to know what I am thinking about the treatment I am taking. So, I thought the PAD is important to fill and safe for me to fill out”* [ID 24, Female, Client].

However, while the PAD helped to fuel confidence in decision-making about care and treatment for sixteen clients, it did not appear to additionally help fifteen clients with feeling more confident about decision-making in other domains. Only three women felt that they had more confidence to make decisions in other domains of life.

*“I cannot take decisions in my life, but treatment is different, because I know what kind of medicine is good for me, what treatment is good for me, I can take decisions on that. But life decisions are different, I always have to depend on my husband and I don’t have anybody else, it is good that I actually consult my husband and decide. I think I can make [treatment] decisions, but it is difficult to make life decisions, I see both as different”* [ID 14, Female, Client].

Two clients felt PADs should only be reserved for people capable of answering questions posed by the PAD facilitator. The majority (16 of 18) felt happy with the opportunity to document what they wanted from care in their PAD, and appreciated that their wishes were inquired about:

*“You asked me some very relevant and important questions in the PAD, which other people don’t ask. If you go to other doctors they want to treat you according to the way they want. And you got some very valuable information on what sort of treatment I would prefer and what treatment I would not.. what sort of doses and medication and what is useful and what is not..Writing the PAD is quite thoughtful, and down the line..it will help me to make some choice in case I need hospitalization or I need to be at a good place.. or need different or more treatment… I think, definitely it could help other people to complete a PAD.. I think a PAD would be helpful. I think it will be helpful if people have a say in their own life”* [ID 21, Male, Client].

Overall, emerging from the interviews, PADs were found to have positive impact in 3 main areas: increased self-confidence, increased motivation to participate in treatment choices, and restoring perceived control over circumstances.

*“I think writing the PAD will help me have control over future treatment, because I wrote it like a will, for my safety in the future. I liked it and think it will help me have control”* [ID 2, Female, Client].

The PAD was mentioned to be important to secure future care, safety and health, and facilitate the process of speaking out about preferences, which was motivating for clients. Specific to treatment, PADs were found to be helpful in highlighting the importance of preventing relapse, and adhering to treatment more than before. The interviews revealed two negative associations with PADs. The first was the PAD served as a reminder of painful memories from past experiences, and the second was scepticism about the value and utility of completing a PAD and whether it would be used in future care. The latter experience was linked to the feeling that the PAD was just a series of ‘answering of questions’, or redundant to document preferences and decisions that would otherwise be allocated to carers or health care professionals.

*“I don’t know if it [the PAD] is important for me. Because I now know that only in the future the PAD might be implemented. I only gave answers because I was asked questions, so I don’t think it is very important”* [ID 8, Male, Client].

## Discussion

The aim of this study was to explore the perspectives of clients and carers to understand the feasibility and utility of psychiatric advance directives in India. To do this, we explored notions of decision-making, knowledge and attitudes towards PADs, and whether completing a PAD had any impact on clients with mental illness.

We found clients generally had high levels of self-efficacy, which is a facilitating factor for completing PADs, as clients can concretise their self-efficacious behaviour and link it to future actions and decisions. Self-efficacy was compromised by the loss of control over decisions and resources. This loss of control appeared to impact the client’s sense of stability, and was exacerbated when experiencing active symptoms of mental illness. In spite of this, we found that many clients remained motivated and able to achieve goals despite the adversity endured throughout their trajectories (e.g. domestic violence, homelessness, poverty). Although this study did not explicitly highlight client trajectories, the Banyan generally caters to populations afflicted with a number of adverse life events such as poverty, homelessness, and violence. In this regard, this population can be seen as representative of a lower socio-economic background compared to a previous study examining the feasibility of PADs in a population with a higher socio-economic status [16].

Furthermore, our findings indicate that service users engage in a number of forms of decision-making (active, passive, collaborative) depending on the situation. In India, decision-making roles (e.g. about food, education and employment) are often situated within pre-determined norms in society. It is thus important to contextualise decision-making and decision-making powers. For example, choosing to give up decision-making powers to a relative in India can be a decision in itself. Therefore it is not that clients do not make decisions, rather, the operationalisation of a decision in some domains is different compared to the context in which PADs were originally developed. Nevertheless, within these pre-defined roles, we observed that some, but not all clients felt they had the space to exercise their decision-making capacity with varying degrees of support. The reasons for reluctance or inability to make decisions remains unclear, thus it is important to find out the barriers to making decisions (i.e. is it that they are unaware of how to demand the right to articulate decisions, reluctance to take responsibility for this decisions) etc. In order to alleviate these barriers, prior to introducing PADs, the dialogue between health care workers, staff, and clients should be opened up.

The finding that carers had a negative view of service user capacity to articulate preferences and decisions demonstrates the importance to work with carers in developing strategies to help support their relative to make decisions, even if only in certain domains. This is in contrast to findings from another study on PAD in India, which found carers to be very supportive of PADs [16]. The role of family in PADs is a delicate one, as carers may influence preferences articulated in a PAD, or may lead to tense relations if the client and carer preferences differ. However, the role of the family is an important one to consider and discovering a way for involving carers in the PAD process constructively is essential.

Overall, our findings show that PADs are promising for some clients, but in order to increase the value of the tool for clients in India and ensure smoother implementation of PADs in practice in light of the new Mental Health Care Bill, it needs to be adapted to better suit the local context. Prior to this, a number of barriers will arise to adaptation and implementation of this tool. First, the current state of the public health system is fragmented, particularly at the systems level. In a pluralistic health context like India, where it is common to concurrently use services from a number of practitioners from different systems of medicine (e.g. faith-based and biomedical practitioners), it is still unclear how a PAD will be coordinated and honoured across multiple care platforms. Our study is based in an organisation emphasising service user involvement, thus clients may have been more open to such a tool, which differs from processes in the public health system. Furthermore, barriers at the professional level could impact PAD uptake in India. The hierarchical structure of doctor-patient relationships in India means that consultations are often dominated by the doctor’s knowledge, and the client is subordinate [17]. In this type of interaction, clients may be reluctant in expressing preferences for fear of insulting the doctor, or doctors may be hesitant to use PADs for fear of compromising their status or power. On a conceptual level, one thread interwoven throughout all interviews was the difficultly of navigating through culturally-constructed concepts and placing these concepts into context. It was apparent across interviews that the struggle to identify with concepts related to PADs made it difficult to see the value and meaning of PADs or link them to future outcomes. We observed a mismatch between local conceptual formulations compared to the values and principles underscoring a PAD (e.g. autonomy) as well as PAD-related outcomes (e.g. quality of life). In order for PADs to become more meaningful and useful for clients a better understanding of cultural formulations, and how these attributions relate to decision-making is required.

There are several limitations of this study. While this study population is representative of clients coming from a low socio-economic background with mental health problems, the standard of care this population received is not representative of the broader help-seeking population. The clients in this study received a good standard of care from a private, non-profit organisation emphasising service user involvement, which may have translated into clients being more positive about services, and made them more receptive and open to the introduction of PADs as compared to other care contexts. Second, there was variation in the way health workers facilitated PADs; at times, the PAD was used more as a checklist during consultations rather than as a tool to encourage decision-making. Providing additional training on different aspects of PADs could alleviate this. Third, relying on translated interviews potentially poses a threat to the validity the results, as there were semantic miscommunications, specifically with regards to concept formulation between English and Tamil. To mitigate this, the translator participated in the PAD training and had regular evaluations and discussions with the research team on interview style and concepts. Finally, there may have been a social desirability bias inherent in the interviews. Social norms in India foster politeness and agreement; thus disagreement is rare, for fear of being disrespectful or fear that negative answers may compromise care.

There are several future research avenues for PADs in India. First, it would be interesting to delineate how PADs can be meaningful in India, and what areas in life are PADs most useful for. Second, it is necessary to understand the skills necessary to enable dialogue between health professionals, carers, and clients to encourage decision-making, especially given the hierarchical relationship between providers and clients. Third, exploring the perceptions of implementing PADs in India from the lens of a health professional is important. Finally, it would be interesting to compare the feasibility of PADs in a public health context, such as a primary health care centre or tertiary government-run hospital.

## Conclusions

In summary, if adapted to suit the local context, the PAD could potentially have meaning and value for some persons with mental illness in India, particularly in situations where the voice of the service user is masked by family or health care professionals. Our findings show preliminary evidence that the PAD could be beneficial for some service users, specifically in increasing self-efficacy and desire to engage more in decision-making. Our exploratory study highlights some intriguing areas for further exploration and consideration by practitioners and researchers, particularly developing strategies for translating the concepts underscoring a PAD to the Indian context.

## Competing interests

The authors declare that they have no competing financial interests. SP is a member of the National Task Force for the National Mental Health Policy, Ministry of Health and Family Welfare, Government of India, and has been involved in the drafting of the new proposed mental health legislation, commissioned by the Ministry of Health and Family Welfare, Government of India.

## Authors’ contributions

LS and SP designed and managed the study. SDMZ and SD collected and analysed the data and contributed to writing the results of the manuscript. LS SDMZ and SD were responsible for data analysis. LS was responsible for drafting the manuscript. LS SP LN JGFB were responsible for conceptualisation of the manuscript. All authors reviewed the manuscript, and all authors have read and agreed on the final manuscript.

## Supplementary Material

Additional file 1 Consent form for completion of psychiatric advance directive (English). Click here for file

Additional file 2 Consent form for completion of psychiatric advance directive (Tamil). Click here for file

Additional file 3 Guide for facilitating a psychiatric advance directive. Click here for file
