# Supplementary material for: Unpacking the psychiatric advance directive in low-resource settings: an exploratory qualitative study in Tamil Nadu, India
Source: Int J Ment Health Syst. 2013 Dec 26;7:29. doi: 10.1186/1752-4458-7-29 (PMC3877945; doi:10.1186/1752-4458-7-29)
Supplement: Additional file 1 — Consent form for completion of psychiatric advance directive (English). [file 1752-4458-7-29-S1.docx]

**Consent Form: Advance directives**

Date: _________________

Please read this document carefully. Your signature is required for participation. You must be at least 18 years of age to give your consent to participate in research. A copy of consent form will be given to you. Your research participation is voluntary, and you have the right to withdraw at any time, without prejudice, should you object to this study, and this will not in any way affect the service you receive from Banyan. You can ask questions and receive explanation about your participation.

Purpose of the study:

This study aims to assess feasibility of writing Psychiatric Advance Directives (PAD) by persons with mental illness and its qualitative aspects.

What is Psychiatric Advance Directive?

Sometimes during an episode of illness, persons with mental illness are not in a position to make decisions about their treatment. A PAD is a tool for recording and implementing the stated preferences of persons with mental illness regarding treatment of their mental illness for the future during such episodes. PADs allow the person to have a say in making treatment choices and nominating a representative well in advance of a time when they may not be able to make decisions. These preferences can be expressed in writing with or without help from health care staff.

Nature of Participation:

If you agree to participate in the study, in the first place, a research person will briefly ask you about your views on “having a say in your own mental health treatment”, followed by completion of a PAD form, wherein, you will need to state your preferences for mental health treatments. If needed, a facilitator can work with you to assist you in writing the form and/or helping you to understand the form. After completing the PAD form, you will again briefly interviewed to discuss your experience of writing a PAD.

Possible risks:

We are only interested in assessing the feasibility of writing a PAD and in studying its qualitative features. The completed PAD form is not a legal document. In no sense, can any completed form be considered as a valid PAD for any type of execution of free will of that individual expressed in his/her PAD. There is also the possibility that talking about treatments may bring back any upsetting memories of past treatment that you may have received.

Possible Benefits:

If you participate in this study, you will be given valuable information about this research, which may be useful to you to write a more comprehensive PAD. It will also help you think a bit more about the kind of treatments that you would rather have in the future and help you to state your preferences more clearly to the health care staff.

Confidentiality:

You will be assigned a code number which will protect your identity. All data will be kept in secured files. All identifying information will be removed from questionnaires as soon as your participation is complete. You will not be identified in any way during the study, or when the study is published.

Opportunities to Question:

You are free to ask any question related to the research on the participation in the study. Co-investigator/other research person/facilitator will try to answer your queries. Any technical questions about this study may be directed to the Principal Investigator.

Opportunities to leave the study:

If you decide now or at any point to withdraw this consent or stop participating, you are free to do so, and this will not affect the treatment you receive at the Banyan. You are free to skip specific questions and continue participating at no penalty.

Declaration of results:

Investigators will inform you of the results of this study, and the facilitators will give a presentation at Banyan.

Your signature/thumb impression below indicates that you voluntarily agree to participate in this study.

Date: __________________

Name of participant: _________________________________________________________

Signature of Participant: _____________________ OR Thumb impression:

Case ID: ________________

Declaration by person obtaining consent

Although we would like you to try to complete the questionnaire, you are not obligated to complete it. Your participation is completely voluntary and you may withdraw from this study at any time without consequence.

No identifying information is recorded with your data. Your responses are completely confidential and will be stored on a secure computer and/or locked in a cabinet in an office. Only personnel will have access to this area. The data from the questionnaires may also be presented in professional journals or at conferences, but any such presentations will be of general findings and will never breach individual confidentiality.

I hereby declare that I have explained to the participant, the nature, purpose, procedure, risks, benefits, alternatives to participation in the study; that I have ensured that s/he has understood it entirely; that I provided him/her ample opportunity to ask pertinent questions; that I have given him/her sufficient time to decide whether or not to participate in the research; that if s/he is participating in this study, s/he is participating voluntarily.

Name of facilitator/health worker: ______________________________________________

Signature of facilitator/health worker: ___________________________________________

Name of research team member:_______________________________________________

Signature of research team member:____________________________________________

Place: ____________________________________________________________________
