# Supplementary material for: Unpacking the psychiatric advance directive in low-resource settings: an exploratory qualitative study in Tamil Nadu, India
Source: Int J Ment Health Syst. 2013 Dec 26;7:29. doi: 10.1186/1752-4458-7-29 (PMC3877945; doi:10.1186/1752-4458-7-29)
Supplement: Additional file 2 — Consent form for completion of psychiatric advance directive (Tamil). [file 1752-4458-7-29-S2.pdf]

ஒப்புதல் படிவம் : நவீன வழிக்காட்டு முறைகள்.

தேதி : ____________________

இந்த படிவத்தை கவனமாக படிக்கவும். இதில் பங்கு பெருவதற்கு உங்களின் கையொப்பம் மிக அவசியம். இந்த ஆயிவில் பங்கு கொள்வதற்கு நீங்கள் ஒப்புதல் அளிக்க பதினெட்டு வயது நிரம்பி இருக்க வேண்டும். இந்த படிவத்தின் ஒரு நகல் உங்களுக்கு வழங்கப்படும். இந்த ஆய்வில் பங்குக்கொள்வது உங்களின் சொந்த விரும்பும், அதுமட்டுமின்றி இதிலிருந்து எந்த நேரத்திலும் நீங்கள் விலகி கொள்ளலாம் அது உங்களின் உரிமை இதில் எந்த நிபந்தனையும் கிடையாது. இதற்கு நீங்கள் எதிர்ப்பும் தெரிவிக்கலாம் அதன் மூலம் பேனியனிலிருந்து உங்களுக்கு கிடைத்து வரும் எந்த ஒது உதவியும் தடைப்படாது. உங்களின் சந்தேகங்களை கேட்டு அதற்கான விளக்கத்தை பெற்று கொள்ளலாம்.

<u>இந்த ஆய்வுக்கான நோக்கம் :</u>

இதன் முக்கிய நோக்கமானது மனநல பாதிக்கப்பட்ட மக்கள் மற்றும் அதன் தர மேம்பாட்டை உளவியல் நவீன வழிக்காட்டு முறையின் (PAD) மூலம் இன்றும் சிறப்பாக எடுத்து செல்வது.

<u>இந்த உளவியல் நவீன வழிக்காட்டு முறைகள் என்றால் என்ன ?</u>

மனநல பாதிக்கப்பட்ட ஒருவர் அந்த காலக் கட்டத்தில் சிகிச்சையைப் பற்றிய சரியான முடிவை எடுக்கும் நிலையில் இருக்கமாட்டார்கள். அதற்காக தான் இந்த நவீன உளவியல் வழிக்காட்டுமுறை என்ற கருவியை பயன்படுத்தி மனநல சம்மந்தப்பட்ட சிகிச்சை முறையை எப்படி தங்களின் விருப்பத்திற்கு ஏற்றவாறு எடுத்துக் கொள்வதற்கு உதவியாக இருக்கும். உளவியல் நவீன வழிக்காட்டு முறை (PAD) பாதிக்கப்பட்ட ஒருவர் சிகிச்சை முறை தேர்ந்தெடுக்க வாய்ப்பு அளிக்கிறது.

அது மட்டுமின்றி தனக்கு ஒரு பிரதிநிதியை நியமித்து முடிவுகளை எடுக்க முடியாத நேரத்தில் அவர்களின் உதவியை பெற்றுக் கொள்ளலாம். இந்த வாய்ப்புகளை எழுத்துப் பூர்வமாகவும் அல்லது அது இல்லாமலே உடல் நல கவனிப்பாளரின் உதவியை நாடலாம்.

எப்படி பங்கு கொள்ளுதல் :

நீங்கள் சம்மதித்து இந்த ஆய்வில் பங்கு கொண்டால் முதலில், இந்த நேர்காணலின் ஆய்வாளர் தற்போது நீங்கள் பெற்று வரும் மனநல சிகிச்சையைப் பற்றி உங்களேயே விவரிக்க சொல்வார். அதன்பின் இந்த உளவியல் நவீன வழிக்காட்டு முறையின் (PAD) படிவத்தை பூர்த்தி செய்ய வேண்டும். அந்த படிவத்தில் உங்களுக்கு தேவையான சிகிச்சை முறையைப் பற்றி குறிப்பிட வேண்டும். தேவைப்பட்டால் நீங்கள் பிரதிநிதியை கொண்டு இந்த படிவத்தைப் பூர்த்தி செய்யலாம். படிவத்தை பூர்த்தி செய்தப்பின் அதன் விவரங்கள் விரிவாக விவாதிக்கப்படும்.

சாத்தியமான எதிர்கூர்கள் :

நாங்கள் இந்த படிவத்தின் சிறப்புகளை ஆராய்ந்து அதன் சிறப்புகள் பற்றி மதிப்பீடு செய்யவே விரும்புகிறோம். இந்த உளவியல் நவீன வழிக்காட்டு முறை (PAD) படிவம் எந்தவித சட்ட பத்திரம் கிடையாது. எல்லா பூர்த்தி செய்யப்பட்ட உளவியல் நவீன வழிக்காட்டு முறை (PAD) படிவம் தனி ஒருவர் தங்களின் (ஆண் , பெண்) சொந்த அனுபவத்தை செல்வதற்கான சட்டபூர்வமான படிவமாக கருதப்படும். இந்த ஆய்வின் முந்தைய சிகிச்சைப்பற்றி கசப்பான அனுபவங்களை பகிர்ந்துக் கொள்ள வேண்டிய சாத்திய கூறு உண்டு.

சாத்தியமான சலுகைகள் :

இந்த ஆய்வில் பங்கு கொள்வதன் மூலம் உங்களுக்கு ஆய்வைப்பற்றி மேலும் சில முக்கிய தகவல்கள் வழங்கப்படும். அதன் மூலம் உளவியல் நவீன

வழிக்காட்டு முறை (PAD) படிவத்தை மேலும் சிறப்பாக பூர்த்தி செய்ய முடியும். அத்தோடு நீங்கள் எந்தவித சிகிச்சை முறை தேர்ந்தெடுக்க ஒரு வாய்ப்பை ஏற்படுத்தும் அது மட்டுமின்றி நீங்கள் விருப்பமுறையை ஆரோக்கிய நல பணியாளரிடம் தெரிவிக்க உதவுகிறது.

நம்பகத்தன்மை :

உங்களுக்கு தனி குறியீட்டு எண் வழங்கப்படும் அனைத்து தகவல்களும் ஒரு பாதுகாப்பான ஆவணமாக வைக்கப்படும். உங்களின் அனைத்து அடையாள தகவல்களும் வினாதொடரிலிருந்து நீக்கப்படும். உங்களின் அடையாளம் ஆய்வின் போதோ அல்லது ஆய்வை வெளியிடும்போதோ தெரிவிக்கப்படமாட்டாது.

கேள்வி கேட்கும் வாய்ப்பு :

இந்த ஆய்வில் பங்கு அளிக்கும் போது நீங்கள் அது சம்மந்தப்பட்ட கேள்விகளை வெளிப்படையாக கேட்கலாம் அதற்கு ஆய்வின் உடன் விசாரணையாளர் / ஆய்வாளர் / ஒருங்கிணைப்பாளர் / உங்கள் கேள்விகளுக்கு பதில் அளிப்பாளர்கள். எந்த ஒரு தொழில் நுட்ப கேள்விகளை முதன்மை விசாரணையாளரிடம் தெரிவிக்கலாம்.

ஆய்விலிருந்து பாதியில் விலகும் வாய்ப்பு

நீங்கள் இதில் பங்கு கொள்வதிலிருந்துதோ அல்லது ஒப்பந்தத்திலிருந்து விலக நினைத்தால் எந்த காலகட்டத்திலும் எந்த நேரத்திலும் விலகிக்கொள்ளலாம். அதன்மூலம் நீங்கள் தற்போது பேணியனிலிருந்து பெற்று சிகிச்சை எந்த விதத்திலும் பாதிக்காது. நீங்கள் நினைத்தால் சில கேள்விகளுக்கு பதில் அளிக்காமல் தவிர்க்கலாம். அத்ததோடு தொடர்ந்து நீங்கள் ஆய்வில் பங்கு கொள்ளலாம். அதற்கு எந்த அபராதமும் கிடையாது.

அது மட்டுமின்றி தனக்கு ஒரு பிரதிநிதியை நியமித்து முடிவுகளை எடுக்க முடியாத நேரத்தில் அவர்களின் உதவியை பெற்றுக் கொள்ளலாம். இந்த வாய்ப்புகளை எழுத்துப் பூர்வமாகவும் அல்லது அது இல்லாமலே உடல் நல கவனிப்பாளரின் உதவியை நாடலாம்.

எப்படி பங்கு கொள்ளுதல் :

நீங்கள் சம்மதித்து இந்த ஆய்வில் பங்கு கொண்டால் முதலில், இந்த நேர்காணலின் ஆய்வாளர் தற்போது நீங்கள் பெற்று வரும் மனநல சிகிச்சையைப் பற்றி உங்களேயே விவரிக்க சொல்வார். அதன்பின் இந்த உளவியல் நவீன வழிக்காட்டு முறையின் (PAD) படிவத்தை பூர்த்தி செய்ய வேண்டும். அந்த படிவத்தில் உங்களுக்கு தேவையான சிகிச்சை முறையைப் பற்றி குறிப்பிட வேண்டும். தேவைப்பட்டால் நீங்கள் பிரதிநிதியை கொண்டு இந்த படிவத்தைப் பூர்த்தி செய்யலாம். படிவத்தை பூர்த்தி செய்தப்பின் அதன் விவரங்கள் விரிவாக விவாதிக்கப்படும்.

சாத்தியமான எதிர்கூர்கள் :

நாங்கள் இந்த படிவத்தின் சிறப்புகளை ஆராய்ந்து அதன் சிறப்புகள் பற்றி மதிப்பீடு செய்யவே விரும்புகிறோம். இந்த உளவியல் நவீன வழிக்காட்டு முறை (PAD) படிவம் எந்தவித சட்ட பத்திரம் கிடையாது. எல்லா பூர்த்தி செய்யப்பட்ட உளவியல் நவீன வழிக்காட்டு முறை (PAD) படிவம் தனி ஒருவர் தங்களின் (ஆண் , பெண்) சொந்த அனுபவத்தை செல்வதற்கான சட்டபூர்வமான படிவமாக கருதப்படும். இந்த ஆய்வின் முந்தைய சிகிச்சைப்பற்றி கசப்பான அனுபவங்களை பகிர்ந்துக் கொள்ள வேண்டிய சாத்திய கூறு உண்டு.

சாத்தியமான சலுகைகள் :

இந்த ஆய்வில் பங்கு கொள்வதன் மூலம் உங்களுக்கு ஆய்வைப்பற்றி மேலும் சில முக்கிய தகவல்கள் வழங்கப்படும். அதன் மூலம் உளவியல் நவீன

வழிக்காட்டு முறை (PAD) படிவத்தை மேலும் சிறப்பாக பூர்த்தி செய்ய முடியும். அத்தோடு நீங்கள் எந்தவித சிகிச்சை முறை தேர்ந்தெடுக்க ஒரு வாய்ப்பை ஏற்படுத்தும் அது மட்டுமின்றி நீங்கள் விருப்பமுறையை ஆரோக்கிய நல பணியாளரிடம் தெரிவிக்க உதவுகிறது.

நம்பகத்தன்மை :

உங்களுக்கு தனி குறியீட்டு எண் வழங்கப்படும் அனைத்து தகவல்களும் ஒரு பாதுகாப்பான ஆவணமாக வைக்கப்படும். உங்களின் அனைத்து அடையாள தகவல்களும் வினாதொடரிலிருந்து நீக்கப்படும். உங்களின் அடையாளம் ஆய்வின் போதோ அல்லது ஆய்வை வெளியிடும்போதோ தெரிவிக்கப்படமாட்டாது.

கேள்வி கேட்கும் வாய்ப்பு :

இந்த ஆய்வில் பங்கு அளிக்கும் போது நீங்கள் அது சம்மந்தப்பட்ட கேள்விகளை வெளிப்படையாக கேட்கலாம் அதற்கு ஆய்வின் உடன் விசாரணையாளர் / ஆய்வாளர் / ஒருங்கிணைப்பாளர் / உங்கள் கேள்விகளுக்கு பதில் அளிப்பாளர்கள். எந்த ஒரு தொழில் நுட்ப கேள்விகளை முதன்மை விசாரணையாளரிடம் தெரிவிக்கலாம்.

ஆய்விலிருந்து பாதியில் விலகும் வாய்ப்பு

நீங்கள் இதில் பங்கு கொள்வதிலிருந்துதோ அல்லது ஒப்பந்தத்திலிருந்து விலக நினைத்தால் எந்த காலகட்டத்திலும் எந்த நேரத்திலும் விலகிக்கொள்ளலாம். அதன்மூலம் நீங்கள் தற்போது பேனியனிலிருந்து பெற்று சிகிச்சை எந்த விதத்திலும் பாதிக்காது. நீங்கள் நினைத்தால் சில கேள்விகளுக்கு பதில் அளிக்காமல் தவிர்க்கலாம். அத்ததோடு தொடர்ந்து நீங்கள் ஆய்வில் பங்கு கொள்ளலாம். அதற்கு எந்த அபராதமும் கிடையாது.

இந்த முடிவுகளின் தீர்மானங்கள் :

விசாரணையாளர்கள் இந்த முடிவுகள் பற்றி தெரிவிப்பார் மற்றும் ஒருங்கிணைப்பாளர் இந்த ஆய்வை பேணியனில் சமர்ப்பிப்பார்.

உங்களின் கையொப்பம் / கைரேகை குறிப்பிடுவதன் மூலம் நீங்கள் தன்னிச்சையாக ஆய்வில் பங்குப் பெறுவதற்கு சம்மதிக்கிறீர்கள்.

தேதி : ____________________

பங்களிப்பாளரின்பெயர்: ____

பங்களிப்பாளரின் கையொப்பம் : ______________________ கைரேகை :

ஆய்வின் அடையாள எண் : __________________

ஒப்புதல் படிவம் கொடுப்பவரின் தீர்மானம்

இந்த வினா தொடரை முழுமையாக முடிக்க நாங்கள் முயற்சி செய்கிறோம், இருந்தாலும் இது கட்டாயாமும் கிடையாது, உங்களின் பங்களிப்பு தான் முக்கியம், அது மட்டும் இல்லாமல் இந்த ஆய்வில் நீங்கள் நினைத்தால் பாதியிலே விலகி கொள்ளலாம் அதற்கு எந்த பாதிப்பும் கிடையாது.

உங்களின் அடையாளம் சம்மந்தப்பட்ட எந்த தகவலும் ஒளிப்பதிவு செய்யப்படவில்லை, உங்களின் அனைத்து பதில்களும் மிக பத்திரமாக தனி ஒரு கனி பொறியில் வைக்கப்படும், அந்த கனி பொறியை அங்கீகரிக்கப்பட்ட ஒருத்தர் மட்டுமே திறக்க அனுமதி உண்டு, இந்த வினா தொடரில் சேகரிக்கபட்ட தகவல்கள் அனைத்தும் ஏதெனும் பொது தொலைநுட்ப செய்தி இதழிலோ அல்லது கருத்து அரங்கத்திலோ சமர்ப்பிக்கப்படும், அப்போது இது சம்பந்தமான தவகல்களே பேசப்படுமே
தவிர்த்து தனி ஒருவரின் அடையாளத்தை வெளிப்படுத்தமாட்டது

நான் இங்கு பிரகட்டனும் செய்வது என்னவென்றால், இந்த ஆய்விற்க்கு தேவையான அனைத்து தகவல்குளும் அதாவது, இந்த ஆய்வின் குணாதிசியம், நோக்கம், இடர்படுதல், சலுகைகள், ஆய்வில் பங்குகொள்ளுவதற்க்கு மாற்று வலி போன்ற தகவல்களை விளக்கமாக அவளுக்கு வழங்கி உள்ளன, அதை அவர்கள் முழுமையாக புரிந்து கொண்டர்கள் என்று உறுதி செய்கிறேன், அதோடு அவளுக்கு கேள்விகளை புரிந்து அதற்க்கான பதில் அளிக்கவோ அல்லது ஆய்வில் இருந்து விலகி கொள்வதற்கோ பொதுமான அவகாசம் வழங்கி இருக்கிறேன், அதோடு இந்த ஆய்வில் பங்கு கொள்வது அவர்களின் சொந்த விருப்பம் ஆகும்

விசாரனையாளரின்/ஆரோக்கியே நல பணியாளர் பெயர்:____________________

விசாரனையாளரின்/ஆரோக்கியே நல பணியாளர் கையொப்பம் : ____________________

ஆய்வு குழுவில் இருப்பவரின் பெயர் :______________________________

ஆய்வு குழுவில் இருப்பவரின் கையொப்பம் : ______________________________

இடம் : : ____
