# Supplementary material for: Unpacking the psychiatric advance directive in low-resource settings: an exploratory qualitative study in Tamil Nadu, India
Source: Int J Ment Health Syst. 2013 Dec 26;7:29. doi: 10.1186/1752-4458-7-29 (PMC3877945; doi:10.1186/1752-4458-7-29)
Supplement: Additional file 3 — Guide for facilitating a psychiatric advance directive. [file 1752-4458-7-29-S3.docx]

**Psychiatric Advance Directive**

Please use this guide to assist the client in writing an advance directive. Note that this is not a checklist, but only a guide to help you act as a facilitator. You can use your own judgement to decide which topics you will and will not discuss with each specific client. And please be aware that there is no right or wrong PAD and that you must not direct the client into giving an answer that you think is best. It is about what the client think is best for him/herself. Your task is to make sure that the client understands what he/she is saying.

I am going to help you to write a Psychiatric Advance Directive. A Psychiatric Advance Directive is a statement of your wishes for treatment if you become ill in the future and are unable to make decisions for yourself and your treatment. You do not have to complete this worksheet in order to make Psychiatric Advance Directives, but you can use these questions to think about some of the decisions you may want to make about your mental health treatment.

**If you become mentally ill and you are unable to take decisions regarding in your treatment, in such situation:**

- Do you want to be taken to a clinic/hospital to see a doctor?
- Do you want to leave this decision to your caregivers? If yes, who would that be(name, relationship)?
- You should be taken to see a doctor if - [Identifying situations according to participant when s/he would need immediate medical help]
- Would you prefer to be treated as an outpatient or an inpatient? Do you agree to get hospitalized if that is recommended by your doctor?
- Do you agree to take treatment/medication in the hospital?
- What type of medication do you prefer or do not prefer?
- Would you prefer medication in the form of [A] Pills [B] Liquids/Syrups [C] Injections? Tick all that apply.

If the participant chooses [A]/ [B] then ask following questions:

- Are you taking any pills/syrups regularly?
- What are the pills/syrups you are currently taking? How often? At what dosages?
- Would you prefer any specific pills/syrups if you were hospitalized? Which medicines?
- Are there any medicines which you would prefer not to take (for example, because it has not helped you in the past, or has caused side-effects)? Would you not take this medication even if doctor(s) recommended?
- Are there any pills/syrups that you are allergic to or you have had bad reactions to? If so, what are these medications you are allergic to?

If the participant chooses [C] then also ask following questions:

- Is there any injection you regularly take? If yes, what is the injection you are currently taking? How often?
- Would you prefer any specific injection(s) if you were hospitalized? Which medications?
- Are there any injection(s) you would not take? Would you not take these injection(s) even if doctor(s) recommended?
- Are there any injection(s) that you are allergic to or you have had bad reactions to? If so, what are these medications you are allergic to?

There is also a type treatment called Electro-Convulsive Therapy (ECT), commonly known as ‘shock therapy’. It is occasionally used in severe episodes of mental illness, primarily depression. Typically, it is used after multiple trials of medications have failed, but may also be used earlier when a rapid response is urgently needed and/or the patient requests. Here, you can write your preferences for shock therapy. If you don’t have a preference, you can write that you want your doctor to decide for you in the future regarding the need for shock therapy. You can also ask that your nominated representative should to be informed and/or gives consent, before shock therapy treatment is administered.

- Do you consider shock therapy as another type of treatment you would prefer? Yes/No

If no, would you not consider having it even if your doctor recommended it?

OR

If yes, Do you want to let your nominated representative take this decision when necessary ?

- If yes, under what circumstances? Describe the circumstances when you want or would consider having shock therapy:
- Do you prefer talking about your problems with some one? Yes / No
- If yes, how frequently would you like to talk to the person, say counsellor?
- Would you like to appoint someone to make decision on your behalf when you are mentally ill?
- Who do you want to appoint?
- Do you agree to discuss with this person you just mentioned your wishes regarding mental health treatment when your mental health is good? Yes / No
- Do you have hesitations or concerns about him/her acting as your nominated representative?

Yes / No
